# Supplementary material for: Cryptosporidium modifies intestinal microvilli through an exported virulence factor
Source: Cell Host Microbe. Author manuscript; Available in PMC 2026 Mar 30. (PMC7618952; doi:10.1016/j.chom.2025.04.001)
Supplement: Supplementary Figures [file EMS213016-supplement-Supplementary_Figures.pdf]

Cell Host & Microbe, Volume *33*

Supplemental information

Cryptosporidium modifies intestinal microvilli through an exported virulence factor

Elena Rodrigues, Mitchell A. Pallett, Lorian C. Straker, Tapoka T. Mkandawire, Katarzyna Sala, Lucy Collinson, and Adam Sateriale

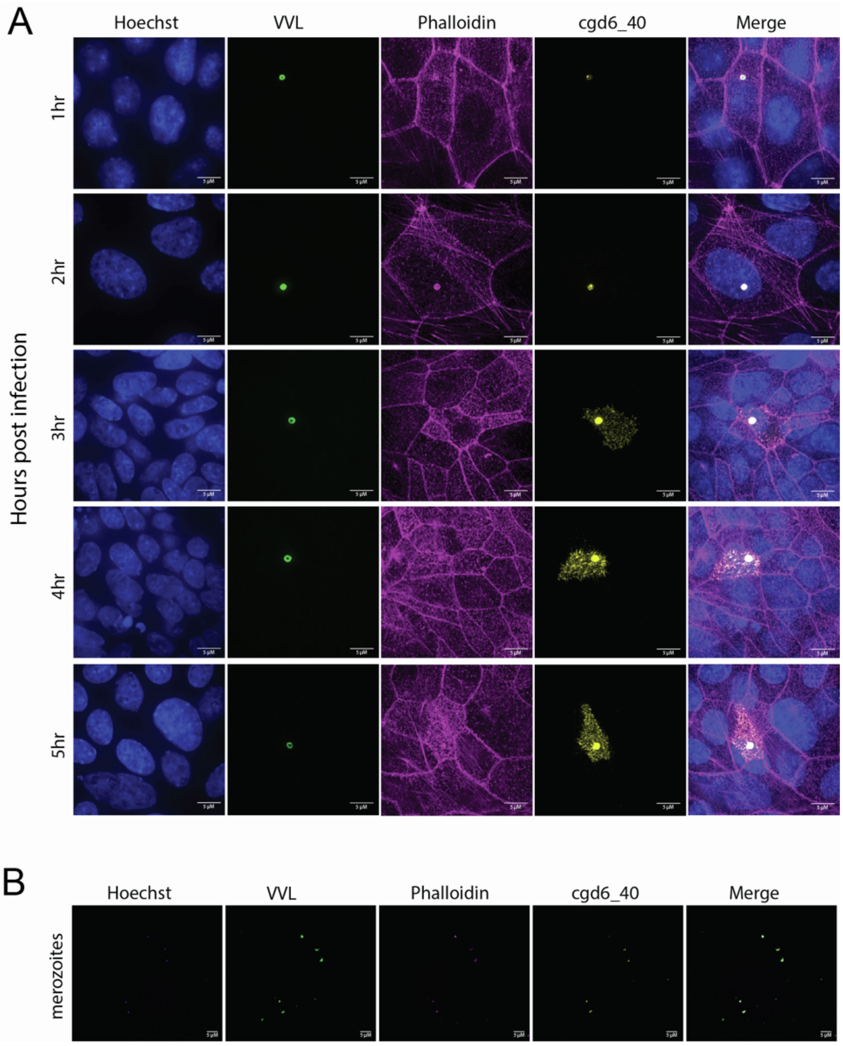

Supplementary Figure S1. Host-export of cgd6_40 following invasion, related to Figure 1. **A)** IFA of epithelial cell (HCT-8) monolayers infected with transgenic cgd6_40-HA parasites. Infections were fixed at one-hour intervals following infection. **B)** Isolated cgd6-40-HA merozoites demonstrating cgd6_40 expression prior to reinvasion.

Predicted Protein Features

Signal peptide | Export motif | Palmitoylation site | Serine repeats

AIUPred - Prediction of Intrinsically Unstructured Proteins

Gabor Erdos & Zsuzsanna Dosztanyi. Nucleic Acids Research V52 W1 2024

MVP1 - cgd6_40

MKFLSNLSIISILAVIAVFYSFTSTESDLASSSYYELSFVKVKCGGGFCKRLRRALCCCSG
GEEEEQPEMVIGNPTDFQHLHSGGLQMRDSDGRLTSRIPSLGTSGPDFSNEGYQSG
DEDNNEGNDDTSSVTSSSSSSSSSSSSSSSSSSSSSSSSGSSSSGSSSSGSSSSGSSS
SSSPPVNGNEYDTKL

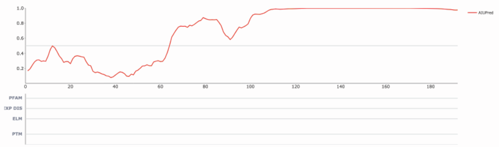

MVP2 - cgd2_390

MSIKKFRSLLTTLVLILFLFNYEHNEQETTNRVVLDHSFLNLRRPSIRKLMRCFGCCRGK
CKKKPSKKKGFTLPPPPARSCLKKTGQNSQSQEGNQGVSPKRVTFDLGNKPRSGIKST
VGGLPSSSSPPLRVMLGLERPDTTKNEDNSEEIETKF

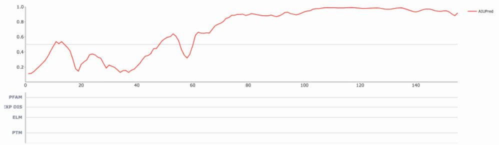

MVP3 - cgd2_400

MTFITNYFYAILLILIINSAFFTINEVHPSEYKFSILQIKAPRCPKLRSLLCCCSASSENEDD
ELQNQNEGAGSGVPVLLAYNPISNTDESNSGGTNGNDEEDPKCCSSSSSSSSSSSSSS
KRKKVDVSKSHPSHPGLYTELLTFQPEVGAXSGFTNTATRFPPSDPDPPEFIPNVRTTT
TTTTRKTETTSNDS

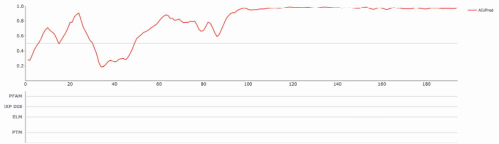

MVP4 - cgd2_410

MQLRNCLILIIFVFSLSFLSFEHRNERDIVVKDRVSFLKLRNGISFGPNLCRRLCCCRGN
GNDLSTQDVVVFAVDNPAFNSNDESSESSSSNSMESVPNPDPSNIPVPSSGPVVSST
SQYSGYSSTSEPIPIPVRPGSNQGQSTPSFPSPCSPSASASKLGFLGMRSPSVSSCVRH
SSSSSSLSSSSTSSSSYSSSSSSSEDEDRETSTNKESGKRGHRGRKTGKKSRSRSKSRSR
SKFKSRSKSRSRSPSPVFKFDEELT

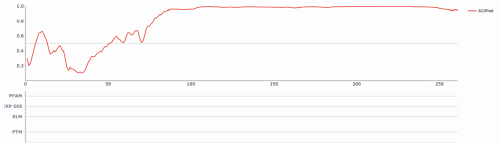

MVP5 - cgd2_450

MNTVKLNFIPLVLCLAFLSNFLGIHDQILSLNTHVDHSLIKLSSPRRFLRRLCCCCSSSC
SSDDSCCSSSSNRGSIEIGNPTQPKHVWSVTGYYGGFGEEMPTEPPRTQEGDGGNE
QSEESEKKSSCCSSSSCKSKKLKLNNGDSNGDSNNDGNSGGNGGRGGPPNPYAGY
TGSSNPTFTTD

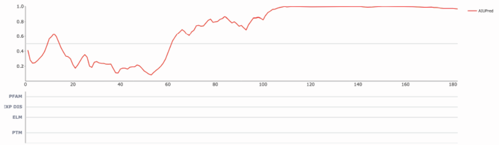

MVP6 - cgd4_10

MKIFKLHLFISFLFLIFQYPSSISFHSNLLSFSFIKVKSPCCKAMRRIRRRLCCCCCSTSCD
SDSEDELNSSSNTSEEQGHSNCQFNPESIDLEVIPPPPPPFPAPQAPVESLSFSPPPSQE
EIGSSFSSEDPDSLPSLPPPPPPVLPKPRRSVLRRVLNI

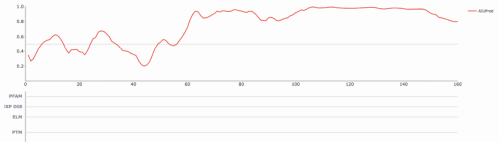

Supplementary Figure S2. Protein feature of the Cryptosporidium parvum MicroVilli Protein family, related to Figure 3. Predicted features on left with signal peptide prediction via CryptoDB[1]. Prediction of intrinsic disorder on right via AIUPred[2].

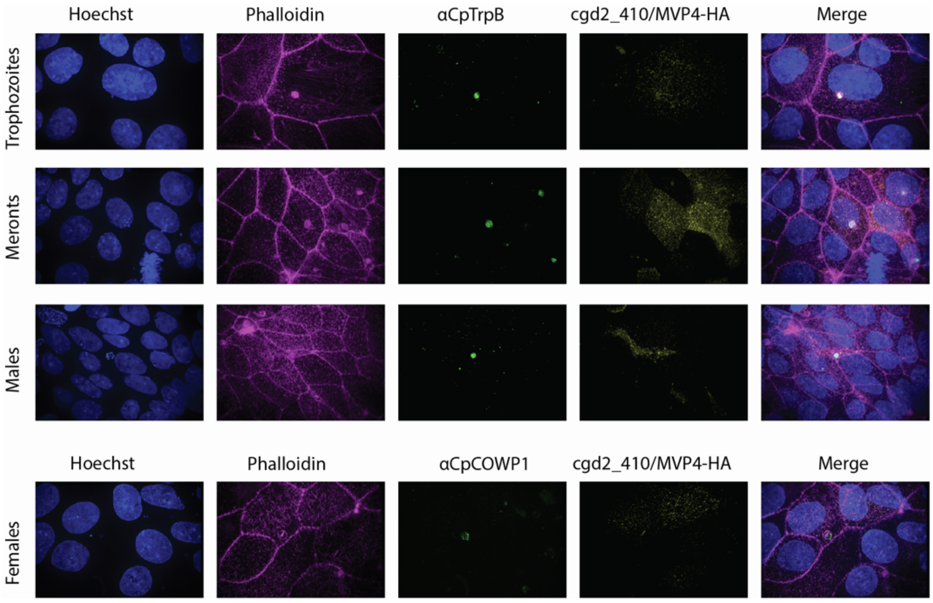

Supplementary Figure S3. Lifecycle expression and localisation of cgd2_410-HA, related to Figure 3. IFA of epithelial cell (HCT-8) monolayers infected with transgenic cgd6_40-HA parasites. Infections were fixed at 6 hours (trophozoites), 24 hours (meronts) and 48 hours (males and females).

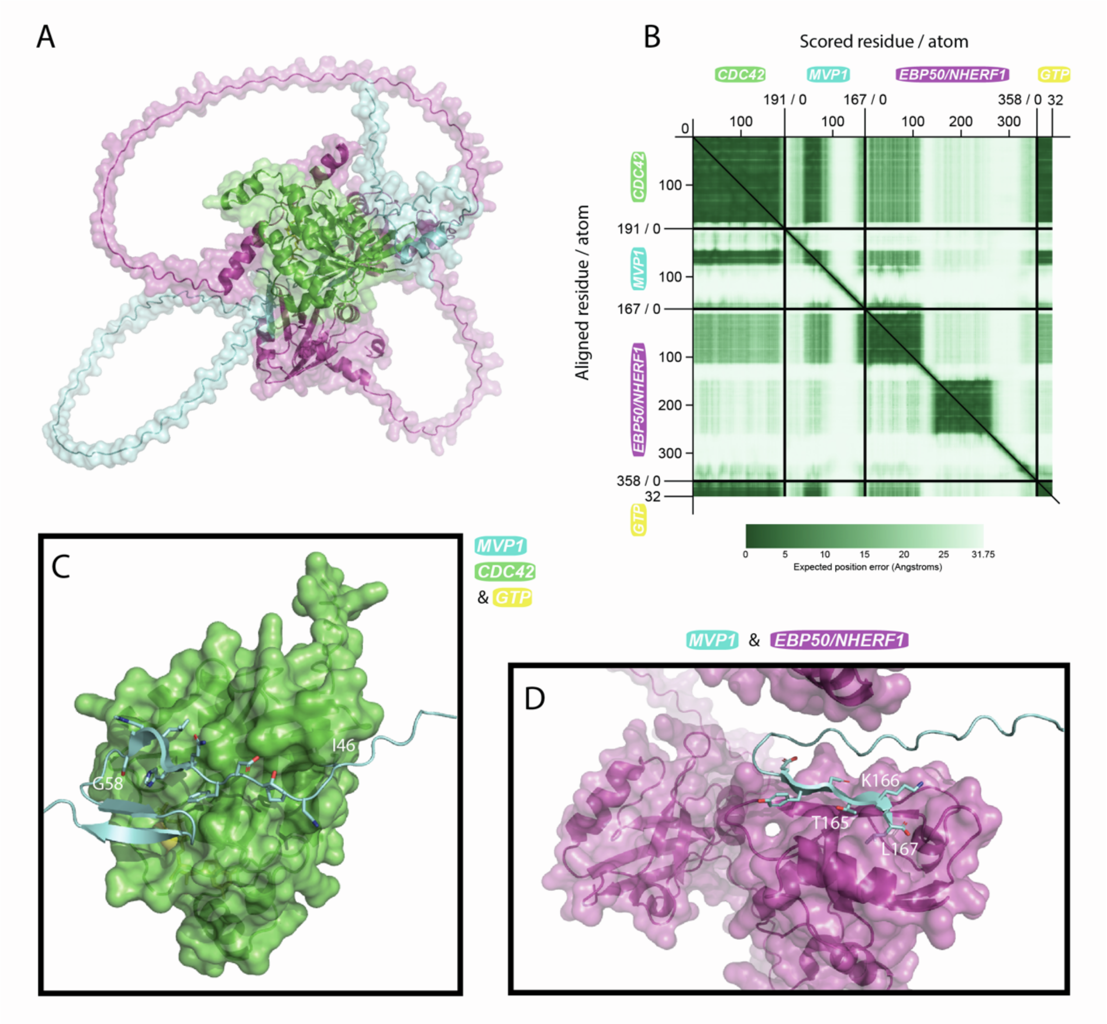

Supplementary Figure S4. Prediction of MVP1 interactions with EBP50 and CDC42, related to Figure 5. Amino acid sequences of MVP1 (cgd6_40 minus signal peptide), human EBP50 (UniProt O14745), human CDC42 (UniProt P60953), and ligand GTP were used for modelling via AlphaFold[3]. Structure visualised via PyMol and PAEviewer[4].

Supplemental Reference List

1. Alvarez-Jarreta, J., Amos, B., Aurrecoechea, C., Bah, S., Barba, M., Barreto, A., Basenko, E.Y., Belnap, R., Blevins, A., Böhme, U., et al. (2024). VEuPathDB: the eukaryotic pathogen, vector and host bioinformatics resource center in 2023. Nucleic Acids Res *52*, D808-D816. 10.1093/nar/gkad1003.
2. Erdős, G., and Dosztányi, Z. (2024). AIUPred: combining energy estimation with deep learning for the enhanced prediction of protein disorder. Nucleic Acids Res *52*, W176-W181. 10.1093/nar/gkae385.
3. Abramson, J., Adler, J., Dunger, J., Evans, R., Green, T., Pritzel, A., Ronneberger, O., Willmore, L., Ballard, A.J., Bambrick, J., et al. (2024). Addendum: Accurate structure prediction of biomolecular interactions with AlphaFold 3. Nature *636*, E4. 10.1038/s41586-024-08416-7.
4. Elfmann, C., and Stülke, J. (2023). PAE viewer: a webserver for the interactive visualization of the predicted aligned error for multimer structure predictions and crosslinks. Nucleic Acids Res *51*, W404-W410. 10.1093/nar/gkad350.
